# Shallow slow earthquakes to decipher future catastrophic earthquakes in the Guerrero seismic gap

**DOI:** 10.1038/s41467-021-24210-9

**Published:** 2021-06-28

**Authors:** R. Plata-Martinez, S. Ide, M. Shinohara, E. S. Garcia, N. Mizuno, L. A. Dominguez, T. Taira, Y. Yamashita, A. Toh, T. Yamada, J. Real, A. Husker, V. M. Cruz-Atienza, Y. Ito

**Affiliations:** 1 Division of Earth and Planetary sciences, Kyoto University, Kyoto, Japan; 2 Department of Earth and Planetary Science, School of Science, The University of Tokyo, Tokyo, Japan; 3 Earthquake Research Institute, Tokyo University, Tokyo, Japan; 4 Research Center for Earthquake Prediction, Disaster Prevention Research Institute, Kyoto University, Kyoto, Japan; 5 Escuela Nacional de Estudios Superiores Unidad Morelia, Universidad Nacional Autónoma de México, Morelia, Michoacán Mexico; 6 UC Berkeley Seismological Lab, University of California, Berkeley, CA USA; 7 Seismology and Volcanology Department, Japan Meteorological Agency, Tokyo, Japan; 8 Instituto de Geofísica, Universidad Nacional Autónoma de México, Ciudad de México, Mexico; 9 Seismological Laboratory, Caltech, Pasadena, CA USA

**Keywords:** Geophysics, Seismology

## Abstract

The Guerrero seismic gap is presumed to be a major source of seismic and tsunami hazard along the Mexican subduction zone. Until recently, there were limited observations at the shallow portion of the plate interface offshore Guerrero, so we deployed instruments there to better characterize the extent of the seismogenic zone. Here we report the discovery of episodic shallow tremors and potential slow slip events in Guerrero offshore. Their distribution, together with that of repeating earthquakes, seismicity, residual gravity and bathymetry, suggest that a portion of the shallow plate interface in the gap undergoes stable slip. This mechanical condition may not only explain the long return period of large earthquakes inside the gap, but also reveals why the rupture from past M < 8 earthquakes on adjacent megathrust segments did not propagate into the gap to result in much larger events. However, dynamic rupture effects could drive one of these nearby earthquakes to break through the entire Guerrero seismic gap.

## Abstract (web summary)

New offshore observations in the Guerrero seismic gap discovered shallow slow earthquakes, which suggest that a portion of the plate interface undergoes stable slip. This may explain the long return period of large earthquakes and why have previous large earthquakes not propagated into the gap.

## Introduction

In recent years, slow earthquakes have been observed at some subduction zones at shallow depths (0–20 km deep) in the updip regions close to the trench^1–3^. Nevertheless, in contrast to downdip slow earthquakes^4^, shallow slow earthquakes are still not well understood. Some representative observations of shallow slow earthquakes come from the Japan Trench, where episodic tremor and slip (ETS) preceded the great Tohoku-Oki 2011, M_w_ 9 earthquake, revealing that slow slip and megathrust earthquakes can coexist at shallow depths of the plate interface^5^. For this reason, the near trench portions of subduction zones should be extensively studied to understand the influence of newly subducted materials on its mechanical properties and assess the actual extent of seismogenic zones, which may produce tsunamigenic and devastating earthquakes.

A proposed earthquake scenario for the Guerrero seismic gap (GG), located along the Pacific coast of Mexico, has been one of a pending rupture of the entire gap capable of generating a large earthquake with M_w_ > 8^6^. An earthquake of this magnitude in the seismic gap could produce ground accelerations in Mexico City twice as high as those experienced during the disastrous M_W_ 8 Michoacán earthquake of 1985^7^, where several hundred buildings collapsed and around 10,000 people died. The associated tsunami could also be catastrophic in large coastal communities such as Acapulco and Zihuatanejo, among many others. Assessing the seismic potential of the GG is therefore one of the most urgent issues worldwide, given the risk to which more than 15 million people in the country’s capital are exposed.

**Fig. 1:**
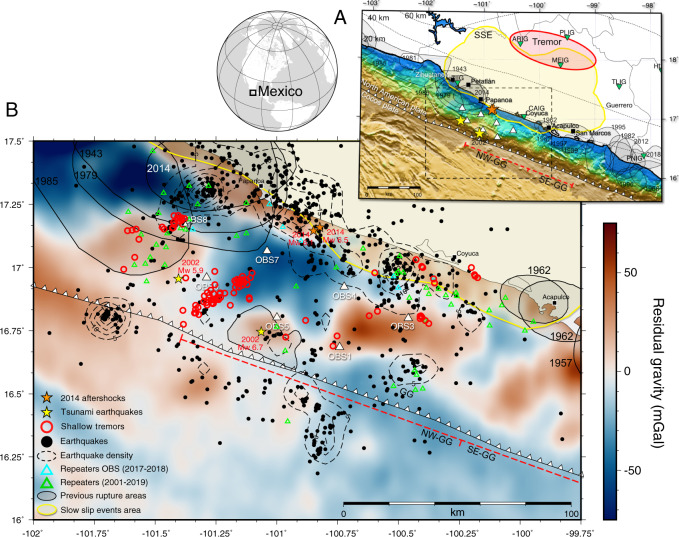
Guerrero subduction zone. **A** White triangles and inverted green triangles show the location of ocean bottom seismometers (OBSs) and land stations, respectively. Grey, yellow and red areas are past earthquake rupture zones^10,58^, SSEs and tremors areas, respectively. Red dashed line is the Guerrero seismic gap, divided into north-west (NW-GG) and south-east (SE-GG) segments. Black dotted lines are contours for the depth of the top of the subducting slab^59^. Black squares are major population centres along the Pacific coast of Mexico. The black dashed square is the area shown in **B**. **B** Residual gravity at the NW-GG. Yellow and orange stars indicate epicentres of the 2002 tsunami earthquakes and aftershocks of the 2014 earthquake, respectively. Red circles, green triangles, cyan triangles and black circles are locations of STs, repeaters (2001–2019), OBS repeaters (2017–2018) and regular earthquakes, respectively. Black dashed lines are contour lines for earthquake density.

The north-west segment of the Guerrero seismic gap (NW-GG), with a length of approximately 140 km (Fig. 1A), has not experienced an earthquake with M > 7 since 1911^8^. The largest seismic events in the NW-GG since then have been two near-trench tsunami earthquakes in 2002 (M_w_ 6.7, M_w_ 5.9)^9,10^ and two aftershocks of the 2014 M_w_ 7.3 Papanoa event with magnitudes M_w_ 6.5 and M_w_ 6.1^11,12^. The Guerrero subduction zone is also prone to slow earthquakes, including some of the largest slow slip events (SSE) in the world, with M_w_ > 7.5, that repeat almost every 4 years^8,13^. Accordingly, the NW-GG accommodates stress aseismically as revealed by geodetic data, suggesting that coupling at the plate interface is approximately 75% lower than that of the bordering segments^14,15^. Since these contributions come from onshore observations, our knowledge of the shallow plate interface regions is limited.

One type of slow earthquake is tectonic tremor, a long duration burst of intermittent seismic signals^16^. Tremors are a good indicator of shear slip at the plate interface and are typically correlated temporally and spatially to SSE in ETS^17^. Offshore observations show that shallow tremor (ST) is also accompanied by SSEs in the weakly coupled shallow plate interface^5,18^. Repeating earthquakes (or repeaters) are the recurrent rupture of small patch-like regions together with surrounding aseismic sliding, making them another useful proxy for aseismic slip^19^. In Guerrero, tremors have been identified at approximately 200 km downdip and 40–50 km depths^20,21^ along the horizontally subducting slab^22^ (Fig. 1A). These tremors describe rapid migration and have been a useful monitoring tool to infer the location and occurrence time of slow slip on the plate interface^23–25^, due to the close interconnection they have with SSE and overpressured fluids at the interface^21,25,26^. All of these observations were carried out using mostly onshore temporary stations and a few permanent stations, limiting tremor detection to selected time periods and regions^27^. So far, shallow slow earthquakes have not been observed at the Mexican segment of the Middle America Trench most likely due to the absence of offshore instrumentation.

Here we analyze the first ever set of offshore observations performed in the GG, corresponding to a one-year period starting from November 2017. An array of seven ocean bottom seismometers (OBS) was deployed inside the NW-GG^28^ (Fig. 1A) to improve monitoring of seismic activity in search of shallow slow earthquakes. The OBS stations were equipped with three component 1 Hz short period sensors and installed at water depths ranging between 980 and 2350 [m]. Their locations were estimated with a mean uncertainty of 2 metres and the data was corrected to remove time shifts on seismic recordings^29^. An envelope correlation method^30^ was used to detect STs as an initial evidence of shallow slow earthquakes in the GG. To explore the marine area with higher spatial resolution, regular earthquakes were also detected using continuous OBS data. Additionally, repeaters were identified using onshore data in a period from 2001 to 2019, and then complemented with offshore data from 2017 to 2018. Based on these results, together with information from residual gravity and residual bathymetry, we seek to answer critical questions such as whether slow earthquakes occur offshore and near the trench, what conditions are required to generate them and what are their characteristics. Most importantly, we seek to improve our understanding of possible future large earthquakes in the Guerrero seismic gap.

## Results

### Seismological observations

**Fig. 2:**
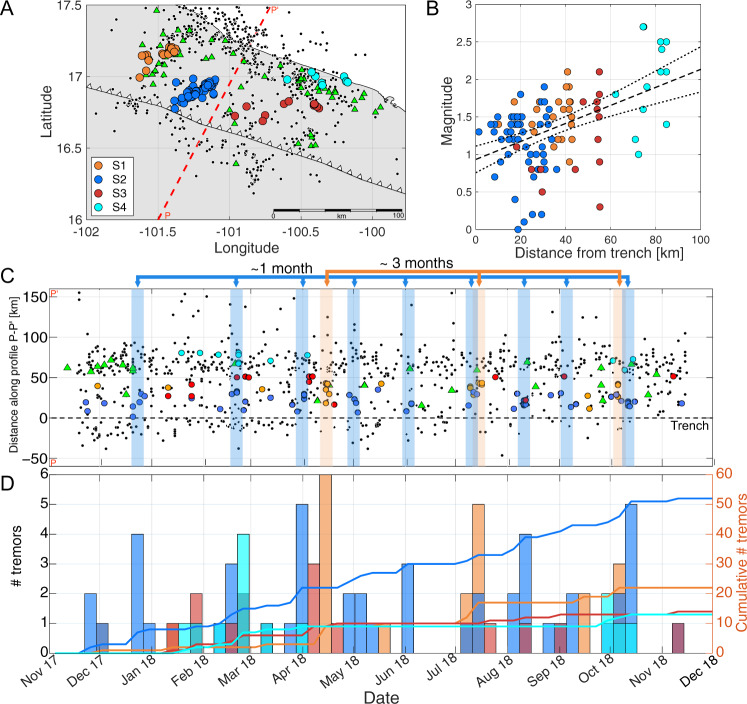
Tremor clustering. **A** Distribution of STs (circles) into four main clusters S1–S4. Green triangles and black circles are repeaters and earthquakes, respectively. Red dash line is profile PP’. **B** Magnitude vs distance from the trench. Solid and dashed lines are the best linear fit and its uncertainty, respectively. Colours and symbols as in **A**. **C** Time plot vs distance along profile P-P’ in **A**. Black dashed line is the location of the trench. Coloured areas are ST episodes in clusters S1 and S2. Colours and symbols as in **A**, showing only events occurring during 2017–2018. **D** Weekly histogram (left axis) and cumulative number of ST in each cluster (right axis). Colours as in **A**.

We detected and located over 100 STs (Fig. 1B) within two kilometres uncertainty in their location (Supplementary Fig. 1). Most of the STs occurred between 10 and 16 km depth (Supplementary Fig. 1), so we assume they rupture at the plate interface as tremors occurring downdip^20,25,26^. However, depth is the least constrained parameter in our locations. No clear ST migration was observed (Fig. 2C) as in regions further downdip^25^, suggesting that ST occur at mechanically isolated locked patches embedded in a weakly coupled plate interface^31^.

Most of STs are located at short distances from the trench (<30 km), while most detected seismicity lies around 60 km away from the trench close to the coast (Fig. 1B). There is a clear trench-perpendicular separation between STs and earthquakes, with a ~20 km-wide region devoid of seismic activity that we refer to as a “silent zone” hereafter. Repeaters coincide with STs in the east and west regions, indicating aseismic slip in these areas.

A least-squares fit with a hypothesis test^32^ confirms a weak correlation between ST magnitude and distance along dip (Fig. 2B). ST magnitude and duration tend to be larger closer to the coast where seismicity intensifies, and coupling is expected to increase. Further observation is still required to confirm this conjecture.

We spatially grouped STs into four main clusters, S1 to S4 (Fig. 2A). Each cluster shows characteristic behaviours and source properties in their magnitude and duration (Supplementary Fig. 2). Clusters S3 and S4 are located at the east side of the study area and have fewer number of tremors. However, S4 (close to the coastline) has the largest magnitude tremors with a mean magnitude of 2.0 ± 0.14. S1 and S2 are located at the west side of the array of OBS stations. These two clusters contain most of the ST in the region with episodes of increased activity. The increased activity comes as episodic tremor bursts with recurrence periods of three months and one month for S1 and S2, respectively (Fig. 2C, D). The episodic ST can be employed as preliminary and first near trench observation of where and when SSE occurred offshore Guerrero^23,24^. Assuming the occurrence of aseismic slip where repeaters and tremors take place, it is most likely that the episodic STs are associated with nearby short-term SSE with one month and three month recurrence periods. Characteristic source properties and different recurrence intervals of STs in each cluster are an indicator of a heterogeneous plate interface regulating the behaviour of tremors.

### Residual gravity and bathymetry

Seafloor geodetic observations at other subduction zones have revealed that subducting relief can increase pore pressure along the megathrust and create a complex system of fractures within the overriding plate^33^ reducing the interplate coupling and generating heterogeneous stress fields at the plate interface^31^. This heterogeneity may lead to a mixture of mechanical conditions controlling macroscopic fault slip and thus enhancing the generation of slow earthquakes^25,34^. Further observations have also shown that the presence of a subducting seamount can lead to the development of a creeping region, capable of producing a diversity of slip behaviours including SSE, tremor or even tsunami earthquakes^35–37^. One way to identify subducting relief at the plate interface is by interpreting residual gravity and residual bathymetry anomalies (RG&BA) which have also been associated with small earthquakes and creep^38^.

In the NW-GG there are some seamounts moving with the oceanic crust towards future subduction (Fig. 1A, Supplementary Fig. 3). Additionally, the 2002 near-trench tsunami earthquake (M_w_ 6.7) overlaps with a positive RG&BA making it possible for both to be related^36^ (Fig. 1, Supplementary Fig. 3). The silent zone covers a trench-parallel transition of a negative-positive RG&BAs, while repeaters and most STs are located over positive RG&BAs. The large positive and negative RG&BA may thus be interpreted as an irregular subducting relief that increases pore pressure, fracturing and decreases coupling, that then determines the mechanical properties of the plate interface likely to generate tsunami earthquakes, STs, repeaters and/or the silent zone.

### Plate interface mechanical model

The spatial distributions of regular earthquakes and STs reveal one of our most interesting observations, which is the silent zone in the centre of the NW-GG (Fig. 1B), referred to above. There are two possibilities to explain the lack of seismicity there. Firstly, this area could be completely locked and thus accumulating strain, so that the probability of a large earthquake initiating there is large. Secondly, it could basically be unlocked and sliding freely with low coupling, thus decreasing the possibility of a large earthquake in the GG. Considering that onshore geodetic data suggest a lack of coupling along the NW-GG^14,15^, together with the inference of aseismic slip from STs and repeaters, it is reasonable to think that the offshore portion of the NW-GG (i.e., the silent zone) better corresponds to the second hypothesis of an almost freely sliding domain. The heterogeneous distribution of STs, repeaters, earthquakes and large variation of RG&BA, however, suggest local changes of mechanical properties at the offshore plate interface that cannot be explained with a simple unlocked model.

Such heterogeneity in the NW-GG strongly suggests that interplate frictional conditions^39^ vary significantly in space. Regions prone to velocity-strengthening and velocity-weakening slip should be present and interact with each other, leading to a variety of coexisting sliding behaviours^11,40^. Therefore, we interpreted our observations to characterize the study area into well-defined regions with similar sliding behaviour and thus frictional conditions.

**Fig. 3:**
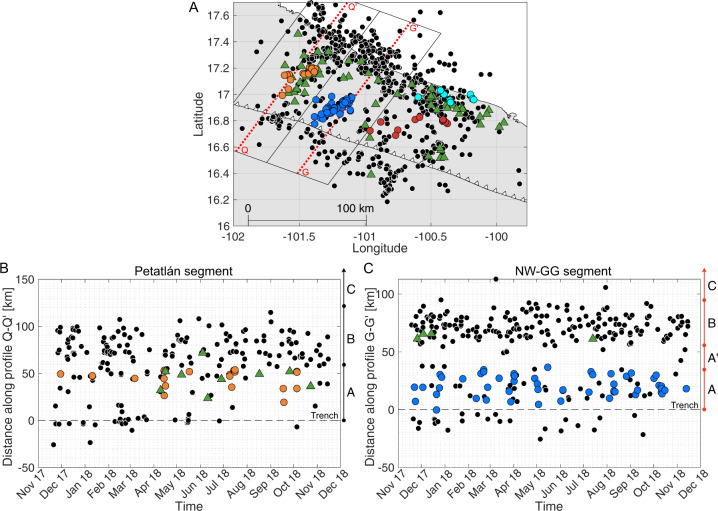
Profiles in the Petatlán and NW-GG segments. **A** Distribution of STs (coloured circles) into four main clusters as in Fig. 2A. Green triangles and black circles are repeaters and earthquakes, respectively. Red dotted lines are profiles G-G’ and Q-Q’ for the NW-GG and Petatlán segment, respectively. Rectangles containing the profiles, restrict the seismic events included into B and C. **B**, **C** Time plot vs distance along profiles Q-Q’ and G-G’ shown in **A**. Black dashed line is the location of the trench. Only seismic events located inside the rectangle areas in **A** and occurring during 2017–2018 are plotted. Colours and symbols as in **A**. Regions described in the main text are shown on the right side of the plots along arrows divided in segments.

**Fig. 4:**
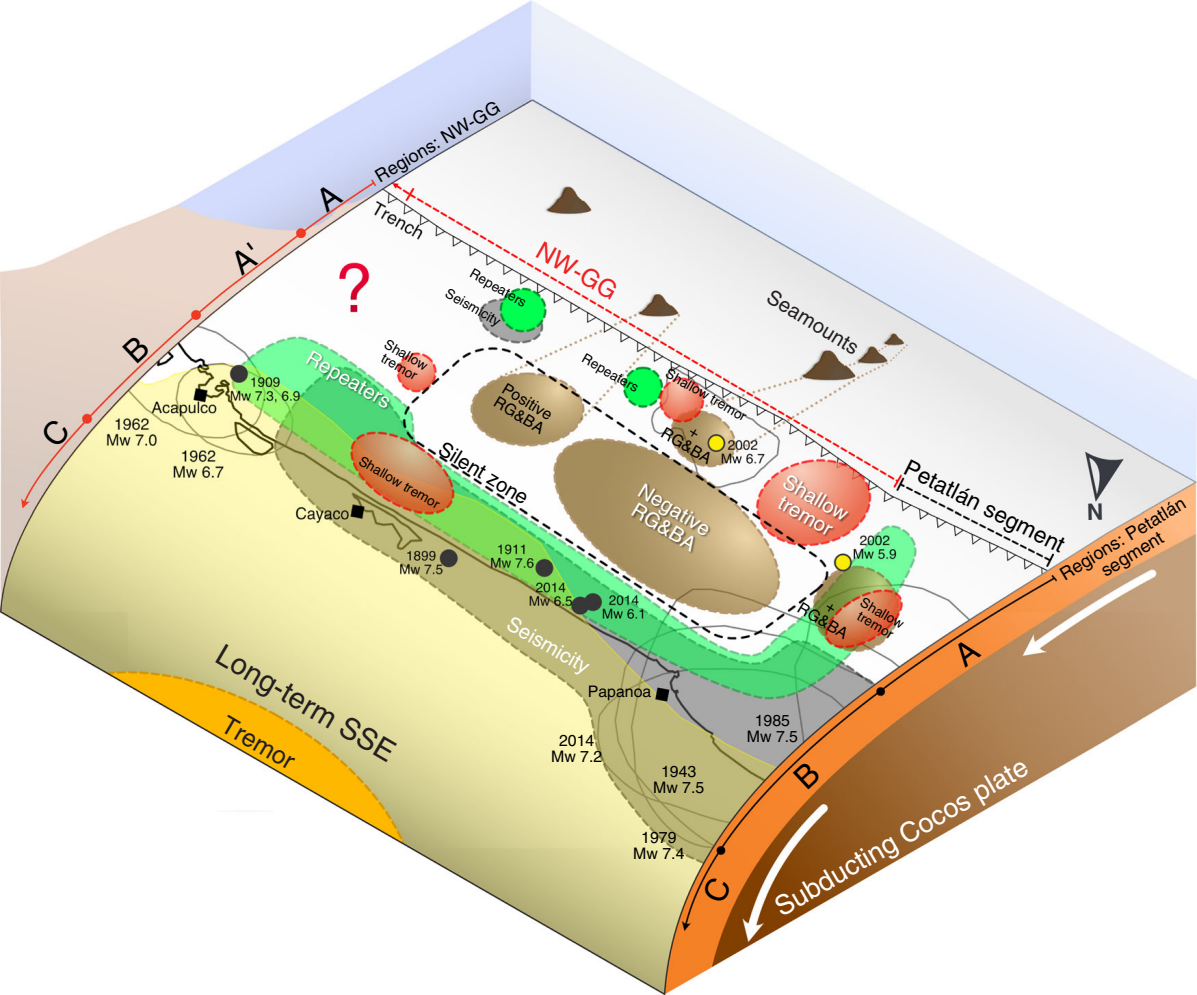
Plate interface conceptual model. Trench is shown with white triangles and the silent zone is shown inside a black dashed line. Red, green, brown, yellow, orange and grey areas are locations of STs, repeaters, RG&BAs, long-term SSE, tremor and seismicity, respectively. Black and yellow circles indicate the epicentres of large earthquakes and tsunami earthquakes, respectively. Estimated rupture areas of previous M > 7 earthquakes are outlined in grey lines. Regions described in the main text for the NW-GG and Petatlán segment are included along arrows divided in segments.

Region A extends from the trench up to 30 km downdip (Fig. 3C), it represents the shallowest interface segment of the NW-GG. In this region we have episodic STs, repeaters, positive RG&BAs and near-trench tsunami earthquakes (Fig. 4). Given the close connection between tremor and SSE^17,21^, the episodic ST could be accompanied by slow slip in the area. Such features would correspond to isolated velocity-weakening asperities within a velocity-strengthening matrix which together comprise a frictionally heterogeneous domain where SSEs can occur and generate episodic ST^40–42^.

Region A’, from 30 to 50 km downdip (Fig. 3C), is formed by the silent zone. It covers an along-strike transition of positive-negative RG&BAs and is surrounded by STs, repeaters, seismicity and rupture areas of M > 7 earthquakes (Fig. 4). The silent zone would correspond to a velocity-strengthening domain where strain is mostly released aseismically, so the possibility of a large earthquake nucleating there is low.

Below the coast, from 50 to 80 km downdip (Fig. 3C), we find Region B, which also contains STs and repeaters, but experiences a larger concentration of small earthquakes and M 7 class earthquakes in the past (Fig. 4). Additionally, large long-term SSEs originating downdip can penetrate this area. Region B is therefore frictionally heterogeneous too, and together with Region A, should contain significant velocity-weakening patches where rapid slip has produced earthquakes with M > 6, such as the tsunami earthquakes, the aftershocks of the 2014 earthquake, or even larger earthquakes like those in the late 19th and early 20th centuries^11^ (Fig. 4). This means that locked patches could eventually generate M > 7 earthquakes despite aseismic slip also occurring periodically. We still have limited data to interpret the south-east segment of regions A, A’ and B (Fig. 4). At farther distances, in Region C, only long-term SSEs and deep tremors occur^13,15,20,21^. Region C has similar slip behaviour as Region A, their difference should be in the mechanisms giving rise to slow earthquakes, with possibly subducted relief, increased pore pressure, sediments and a complex system of fractures facilitating slow earthquakes in Region A^43^.

In addition to the along-dip variations, there is also a clear along-strike change in the mechanical properties between the NW-GG and the adjacent Petatlán segment to the west (Fig. 4). The boundary separating these two segments is outlined by the abrupt end of the silent zone to the west (close to Papanoa) and beyond which STs, repeaters and large earthquakes are more frequent.

In the Petatlán segment, we distinguish three trench-parallel regions (Fig. 3B). From the trench and up to 45 km downdip we find repeaters, ST and possibly SSE, similarly to Region A inside the NW-GG. Slow earthquakes in here may define a transition zone^1^ between the velocity-strengthening silent zone of the NW-GG and an adjacent velocity-weakening segment offshore Petatlán. Further downdip, Region A changes into Region B, a velocity-weakening domain which is absent of STs but seismically active, with repeaters and large earthquakes (M > 7) occurring every ~35 years. At deeper areas we find the same behaviour as in Region C at the NW-GG, where long-term SSEs take place. One of the most important overall differences between the NW-GG and the Petatlán segment is that the former seems to be dominated by mechanically unlocked conditions, which makes the initiation of a large earthquake (M > 8) there less likely.

## Discussion

Many large earthquakes (M > 7) have taken place in the Petatlán segment (Fig. 4); interestingly, these earthquakes have stopped propagating when entering the NW-GG. It is possible that the silent zone acts as a barrier stopping large earthquakes from becoming much larger and rupturing the NW-GG. To do so they will need to continue rupturing across the large velocity-strengthening region in the silent zone. A similar situation is reported at the shallow plate interface of central Peru, where a low coupling and velocity-strengthening region, associated with the subducting Nazca ridge, acts as a barrier for larger earthquakes (M > 8)^44^.

Subducting relief interpreted from RG&BAs in the region could have created highly heterogeneous frictional conditions that contribute to generate the conditions for slow slip, such as episodic STs, possible short-term SSEs repeaters and/or a creeping silent zone. All of our observations indicate that the central portion of the NW-GG is dominated by a weakly coupled domain. Weak coupling, offshore and onshore, explains the long recurrence period of large earthquakes in the GG^45^. This implies that the initiation of a M > 8 earthquake in the NW-GG is less likely to occur as previously expected; however, the risk of a rupture of the GG does not disappear. Stress loading around the creeping silent zone offshore the NW-GG may facilitate a sufficiently large earthquake approaching from an adjacent segment to propagate through the gap driven by dynamic effects, including the cascading rupture of nearby locked patches below the coast, where M > 7 ruptures have occurred in the past. Comparable circumstances where subducted seamounts, seismic and aseismic events coexist in the same interface region has already been described in the Japan Trench^46^; for these reasons, disaster prevention efforts should not be reduced in Guerrero. New offshore and onshore observations, together with physics-based source modelling, could validate our predictions, which are important for further development of seismic risk mitigation.

## Method

### Tremor detections

Tremor detection and location were done following a modified envelope correlation method based on maximum-likelihood^30^. Envelope waveforms were estimated by (1) band‐pass filtering continuous velocity data between 2 and 8 Hz, (2) squaring, (3) low pass filtering at 0.2 Hz and (4) resampling at 1 Hz. Tremor detections were done using 300 s time windows with 150 s time steps and a detection threshold for cross correlation coefficient between stations of 0.6^47^.

Localization of tremors was done considering a normalized envelope waveform ${w}_{i}\left(t\right)$ for all components at all stations^30^.

1 $${w}_{i}\left(t\right)=\frac{{w}_{i}^{{\prime} }(t)-\bar{{w}_{i}^{{\prime} }}}{\sqrt{{\sum }_{k=1}^{{N}_{t}}{\left({w}_{i}^{{\prime} }({t}_{k})-\bar{{w}_{i}^{{\prime} }}\right)}^{2}}},$$

where the sub index $i$ represents the $i$-th component, ${w}_{i}^{{\prime} }(t)$ is the original envelope waveform, $\bar{{w}_{i}^{{\prime} }}$ is the temporal mean, ${N}_{t}$ is the number of time samples and ${t}_{k}$ is the $k$-th time step. ${w}_{i}\left(t\right)$ can also be defined by assuming a source envelope which is a common template waveform $w(t)$ at all stations, but shifted in time considering a travel time ${\triangle t}_{i}(x)$ between source and position $x$, plus a Gaussian error with a distribution $N(0,{\sigma }_{i}^{2})$,

2 $${w}_{i}\left(t+{\triangle t}_{i}(x)\right)=w\left(t\right)+{e}_{i}(t+{\triangle t}_{i}(x)).$$

With these assumptions we can develop the mathematical expressions to maximize the likelihood of finding the observed envelopes from the combination of a common template waveform $w\left(t\right)$ and travel times to position $x$. The maximum likelihood problem can be solved as a summation of cross-correlations weighted by the error variance. We then maximize the averaged of the weighted cross-correlations (ACC),

3 $${ACC}\left(x\right)=\frac{{\sum }_{(i,j)}{\sum }_{k=1}^{{N}_{t}}\frac{{w}_{i}\left({t}_{k}+{\triangle t}_{i}(x)\right){w}_{j}\left({t}_{k}+{\triangle t}_{j}(x)\right)}{{\sigma }_{i}^{2}{\sigma }_{j}^{2}}}{{\sum }_{(i,j)}\frac{1}{{\sigma }_{i}^{2}{\sigma }_{j}^{2}}}.$$

The weight, or error variance, is calculated based in the similarity of the template and observed waveforms. This method is looking for the best tremor location ${x}^{{best}}$, that will maximize ACC, by calculating travel times to any position $x$. Travel times are calculated using ray theory and a 1D velocity model for the Guerrero region^48^.

The maximization problem is solved using a sequence of two steps; first, a grid search with a fixed 10 km depth considering local maxima, and second, a gradient method search^49^ (CCSA) to improve hypocentre locations. The grid search is taking into account local maxima because each of these will come as a result of more than one detection inside the same time window; therefore, this method is able to detect more than one event inside the same time window. Finally, the gradient method uses all the local maxima from the grid search as initial values to refine the location.

To remove outliers, the acquired detections must validate two established conditions. Firstly, cross-correlation coefficients must be larger than 0.6 as initially postulated. However, during the maximization procedure the correlation value could decrease, so this condition must be verified. Secondly, the similarity between envelope and template waveform (Eq. 2) must be good, so if their correlation value is below 0.4, envelopes are rejected. Once outliers are excluded, the localization procedure is repeated starting from the gradient method and continues until no outliers are found. Standard deviation of the final location is estimated using bootstrap, and results with more than two kilometres of error are also excluded.

It is noteworthy to point out that the velocity structure used in tremor location does not include a slow sedimentary layer located at the ocean floor, where OBS are situated at. This will produce a common bias, in which estimated locations should be deeper than expected. This means that depth is the less constrained parameter in our locations.

Source parameters for tremors are estimated using the original envelope waveform. The average of seismic energy rate can be expressed as

4 $${\dot{E}}_{s}\left(t\right)=4\pi \rho \beta \frac{{\sum }_{(i,j)}\left(\frac{{w}_{i}^{{\prime} 2}\left(t+{\triangle t}_{i}({x}^{{best}})\right){R}_{i}^{2}}{{\sigma }_{i}^{2}}+\frac{{w}_{j}^{{\prime} 2}\left(t+{\triangle t}_{j}({x}^{{best}})\right){R}_{j}^{2}}{{\sigma }_{j}^{2}}\right)}{{\sum }_{(i,j)}\left(\frac{1}{{\sigma }_{i}^{2}}+\frac{1}{{\sigma }_{j}^{2}}\right)}.$$

where ${R}_{i}$ is the hypocentral distance to stations, $\rho =3000$ kg/m^3^ is density and $\beta =2.8$ km/s is shear wave velocity. The time when the averaged seismic energy gets to its maximum will be the hypocentral time and duration will be equal to the time required to get one quarter of the maximum energy. Energy magnitude is estimated with ${M}_{e}=\frac{{\log }\left(\int {\dot{E}}_{s}(t){dt}\right)-4.4}{1.5}$^50^.

### Earthquake detections

Continuous OBS data was also used to detect small local seismicity and monitor in detail seismic behaviour at the he marine area. Earthquake detections were done using an automatic method of a short time window over a long-time window (STA/LTA), with a ratio of 2 in at least three stations to trigger a detection. The STA/LTA threshold was determined empirically by visually detecting 74 earthquakes a priori. LTA and STA windows had 10- and 0.3-seconds length, respectively. A total of 4303 events were detected by the STA/LTA method. To distinguish earthquakes from noise we used an automatic picking^51^, to pick *P* and *S* waves and discard those detections where no phase was found. A second stage of visual inspection to avoid any false detections was done. Finally, a new manual picking of *P* and *S* waves was done to a total of 848 events. Location was done following a maximum likelihood method^52^ and using a 1D velocity structure for Guerrero^49^. Density of earthquakes was estimated using a grid covering the complete study area with square elements of 0.1 degrees length. A bivariate histogram was calculated with the number of earthquakes in each element of the grid.

We compared our OBS earthquake catalogue with an alternative catalogue reported by the National Seismological Service (SSN)^53^, from inland stations and earthquake detections since 1908 (Supplementary Fig. 4, Supplementary Fig. 5). For the time period of interest (2017/11/01- 2018/12/01), the SSN catalogue reported a total of 1074 earthquakes in the Guerrero seismic gap region. The OBS and SSN catalogues have 298 common earthquakes that are included in both catalogues. Additionally, the OBS catalogue has 518 new earthquakes not reported by SSN (Supplementary Fig. 5B). Even when the locations of events common to the SSN and OBS catalogues are similar with good correlation values (Supplementary Fig. 4), locations from SSN tend to have a north-west shift with respect to the OBS locations (Supplementary Fig. 5B). These systematic differences must be due to differences in velocity models used for locations, since SSN uses a simple 5 layers 1D velocity model. Moreover, the two networks (offshore and inland) are configured independent of each other and will have difficulty in precisely locating earthquakes occurring outside their range, contributing to a systematic offset.

Earthquake distribution for both catalogues was compared (Supplementary Fig. 5A). The distribution of OBS earthquakes and SSN earthquakes is equivalent. With this we can constrain the earthquake distribution offshore the Guerrero seismic gap with additional certainty. From these extra enquiries, the observations reported in this research are not modified and become better supported. Thus, we can confirm there is no significant artifact that could modify our conclusion and that detectability of reported earthquakes is sufficient to support our conclusions.

### Repeaters

We searched for repeating earthquakes along the trench by analyzing 440,655 waveforms from 13 permanent stations from the SSN^53^ corresponding to 75,567 earthquakes recorded between 2001 though 2019. Furthermore, we analyzed the one-year data from the OBS network, using the ad hoc catalogue described in the previous section. To classify events as repeating earthquakes, we computed the correlation coefficient (CC) and spectral coherency (COH) for all pairs of closely located events (<100 km). The CC and COH was estimated in a 25-s time window starting at the onset of the P-wave arrival in a frequency band of 1–8 Hz and 4–16 Hz for the permanent stations and OBS stations, respectively. We used two different frequency bands to provide a higher signal to noise ratio for the OBS stations which are located in a nosier environment. Sequences were formed by linking pairs of events with a COH and CC higher than 95% for at least two or more stations either permanent or OBS. Clusters of repeaters were formed using hierarchical clustering with “single” method provided from SciPy library. Hence, we found along the GG, a set of 51 sequences consisting of 2 to 4 repeating earthquakes each; the magnitude of these events ranges from 3.4 to 4.5 using the onshore permanent stations from 2001 through 2019. By means of the OBS network, an additional set of 7 sequences was detected with magnitudes between 2.7 and 3.8 with short burst-type recurrence time (<days, month) during the survey period (2017–2018).

### Residual gravity and bathymetry

We used global compilations of marine gravity anomalies and bathymetry data to generate grids of residual gravity and bathymetry^38,54^ covering a region of the Middle America subduction zone offshore the Pacific coast of Mexico. Analyzing residual gravity anomalies and residual bathymetry allows us to discern small-scale local features on the forearc because the broader regional signal associated with subduction has been removed. In order to calculate the residual gravity, we used version 29.1 of the Global Gravity grid^55^ developed and distributed by the Scripps Institution of Oceanography, University of California, San Diego. This global gravity model is provided at 1-arc minute resolution and is constructed using measurements of the sea surface slope collected by several satellite altimeters over separate geodetic missions conducted in the years between 1985 and 2019. We extracted trench-perpendicular profiles every ~25 kilometres along a ~1500-kilometre-long segment of the Middle America subduction zone. The gravity anomaly data (given in units of mGal) along the profiles were then stacked. We then subtracted the trench-perpendicular average gravity anomaly from the original grid to obtain the residual gravity anomalies, which show both positive and negative values in the forearc region above the Guerrero seismic gap. Meanwhile, in order to calculate the residual bathymetry, we requested gridded data from the Global Multi-Resolution Topography (GMRT) Synthesis^56^ at 7.5-arc second resolution. Offshore, the GMRT grid combines data from multiple sources, including publicly available high-resolution multibeam sonar swath surveys and the General Bathymetric Chart of the Oceans^57^. We followed a similar procedure to the processing of the residual gravity in extracting profiles of elevation data, averaging these, and then subtracting this trench-perpendicular average to finally obtain the residual bathymetry (for offshore regions) and residual topography (on land).

From bathymetry data (Fig. 1A) and residual bathymetry data (Supplementary Fig. 3), seamounts can be found in front of the Guerrero seismic gap, at approximately 100 km away from the coast in between longitudes −100.5˚, −100.0 and latitudes 15.7˚, 16.5˚. On the incoming plate these seamounts have basal widths ranging from 10 to 20 kilometres and rising at least several hundred metres above the surrounding seafloor. Seamounts are moving with the oceanic crust towards future subduction. Areas with positive values in the residual gravity are found landward of seamounts on the incoming Cocos Plate (Supplementary Fig. 3). This suggests that some of the positive residual anomalies that share these features could be associated with subducting seamount chains underneath the forearc. Meanwhile, some of the negative residual features are coincident with basins based on interpretation of the seafloor morphology. The gravity data are more directly related to the roughness of subducting topography since it is more sensitive to the structure of the rock basement, whereas the bathymetry measurements are affected by sediment infill or erosional processes occurring on the upper plate.

## Supplementary information

Supplementary Information

Description of Additional Supplementary Files

Supplementary Data 1

Supplementary Data 2

Supplementary Data 3

## Data Availability

Data set of tremors, earthquakes and repeaters are available in Supplementary Information. Codes are available upon request to the correspondence author R. P.-M. (plata.omar.48e.@st.kyoto-u.ac.jp). Ocean bottom seismometers data from SATREPS-UNAM project are subjected to restrictions policies until March 2025. Inland broadband data is available from SSN at: www.ssn.unam.mx.
