# Supplementary material for: Shallow slow earthquakes to decipher future catastrophic earthquakes in the Guerrero seismic gap
Source: Nat Commun. 2021 Jun 28;12:3976. doi: 10.1038/s41467-021-24210-9 (PMC8239025; doi:10.1038/s41467-021-24210-9)
Supplement: Supplementary file 1 — Supplementary Information [file 41467_2021_24210_MOESM1_ESM.pdf]

Supplementary Information for:

Shallow slow earthquakes to decipher future catastrophic earthquakes in the Guerrero seismic gap.

R. Plata-Martinez[1], S. Ide[2], M. Shinohara[3], E. S. Garcia[4], N. Mizuno[2], L. A. Dominguez[5], T. Taira[6], Y. Yamashita[4], A. Toh[2], T. Yamada[3, 7], J. Real[8], A. Husker[9], V. M. Cruz-Atienza[8], Y. Ito[4].

[1] Division of Earth and Planetary sciences, Kyoto University.

[2] Department of Earth and Planetary Science, School of Science, The University of Tokyo.

[3] Earthquake Research Institute, Tokyo University.

[4] Research Centre for Earthquake Prediction, Disaster Prevention Research Institute, Kyoto University.

[5] Escuela Nacional de Estudios Superiores Unidad Morelia, UNAM

[6] UC Berkeley Seismological Lab, University of California.

[7] Seismology and Volcanology Department, Japan Meteorological Agency.

[8] Instituto de Geofísica, UNAM.

[9] Seismological Laboratory, Caltech.

Supplementary Note 1: Spectra estimation

Spectra of seismic waveforms were estimated for regular earthquakes and tremors (Supplementary Fig. 6C). We manually selected time windows including signals from earthquakes or tremors in the two horizontal components of all stations and then estimated displacement spectra in frequency domain. A characteristic spectrum for each event was estimated with the geometrical mean of all observations. Spectra were not corrected by effects from path attenuation and site effect.

Supplementary Note 2: Waveform analysis

The described method for tremor detection acts efficiently, even in the instance where more than one event is located in a same time window. However, it cannot differentiate between tremors, earthquakes, and other seismic signals (e.g., T-phase). A visual inspection of spectrograms and waveforms, for every detection was required to isolate tremors from other type of seismic events. Waveforms of detections were visually evaluated using two different bandpass filters (between 2-8 Hz and 10-30 Hz). Lastly, spectral analysis was used to fully differentiate tremors from regular earthquakes.

For regular earthquakes waveforms and spectrograms have a clear impulsive *P* and *S* waves arrivals and energy at higher frequencies (>10 Hz). Waveforms using both band pass filters (2-8 Hz and 10-30 Hz), have a sharp signal that loses energy rapidly as it travels through the stations array (Supplementary Fig. 6).

On the other hand, tremors have an emergent signal with no clear *P* or *S* phase arrivals. In the spectrogram, energy is concentrated below 10 Hz and disperse in a larger time window (Supplementary Fig. 6). Waveforms vanish when using a bandpass filter between 10-30 Hz but is easily visible when using 2-8 Hz and is similar to other waveforms of shallow tremors observed with OBS in other subductions zones[1, 2, 3].

Tremor seismic signal is clear only at OBS stations located close to the source, and as their seismic waves travel to further stations they attenuated rapidly (Supplementary Fig. 7). Comparing peak ground velocities of detection's signal filtered between 2 – 8 Hz vs distance between source and stations, show that signal attenuates during the first 45 km (Extended Data Figs. 7, 8). At distances beyond this, most signal is lost.

As a comparison of tremor and earthquake waveforms, spectrograms and spectra are shown (Supplementary Fig. 6). Spectra for earthquakes and tremors undergo large attenuation starting from a frequency around 9-10 Hz. A portion of this attenuation comes from the sharp bathymetry of the region, yielding in a small quality factor, Q. Additionally, site effects from the marine environment have not been removed from the spectra. Supplementary Fig. 6C shows a comparison of displacement spectrum of a tremor, a nearby earthquake and background noise recorded in a time window previous to the tremor occurrence. These two events are also shown in a normalized value, of the ratio of the spectrum and their respective background noise levels (Supplementary Fig. 2D). We can only interpret some source characteristics in a reduced range of frequencies between 0.9 and 10 Hz, in which signal to noise ratio is higher and spectra perform differently from background noise. Below this frequency, noise levels are high and above it, spectra undergo large attenuation. Bellow 10 Hz the spectra of earthquakes have a flat progression similar to an ω^{-2} Brune model[4], or close to an ω^{-1} slope. On the other hand, tremor has lower spectral levels, a continuous drop closer to or even steeper than an ω^{-2} slope and does not have energy at frequencies above 10 Hz.

T-phase could also be misclassified as tectonic tremors[5], so it is important to identify any possible t-phase in our detection lists. Typically, T-phase happens only after large earthquakes located under the ocean floor. Their waveform travels slowly at approximately 1.5 km/s (sound velocity

in water) and have energy at frequencies higher than 10 Hz. As T-phase seismo-acoustic waves travel through the OBS array they display negligible attenuation and are hardly visible at inland stations near the coast. With these characteristics it is possible to distinguish T-phase from tremors. Tremor detections times were compared to possible T-phase travel times coming from every earthquake, reported at the NEIC catalogue, that could produce T-phase signals in our OBS array.

Supplementary Note 3: Tremor clustering

We grouped tremors based on their locations into 4 clusters using a K-means algorithm[6]. To determine the number of clusters we employed silhouette diagrams[7]. Silhouette values are used to measure how similar an observation is to the other elements within the same cluster as well as other clusters. A silhouette value close to 1, means there is a good similarity, and that the clustering solution is good. We estimated silhouette values for each ST and used the mean of the silhouette values as a parameter to decide what will be the ideal number of clusters to use. A comparison between silhouette diagrams for 3 and 4 clusters is shown in Supplementary Fig. 9. The silhouette values perform better for 4 clusters with a mean of 0.833, compared to 3 clusters with a mean value of 0.706. The same procedure was done considering 2, 5, 6 and 7 clusters. Four clusters achieved the best results, implying that 4 clusters characterise our data properly.

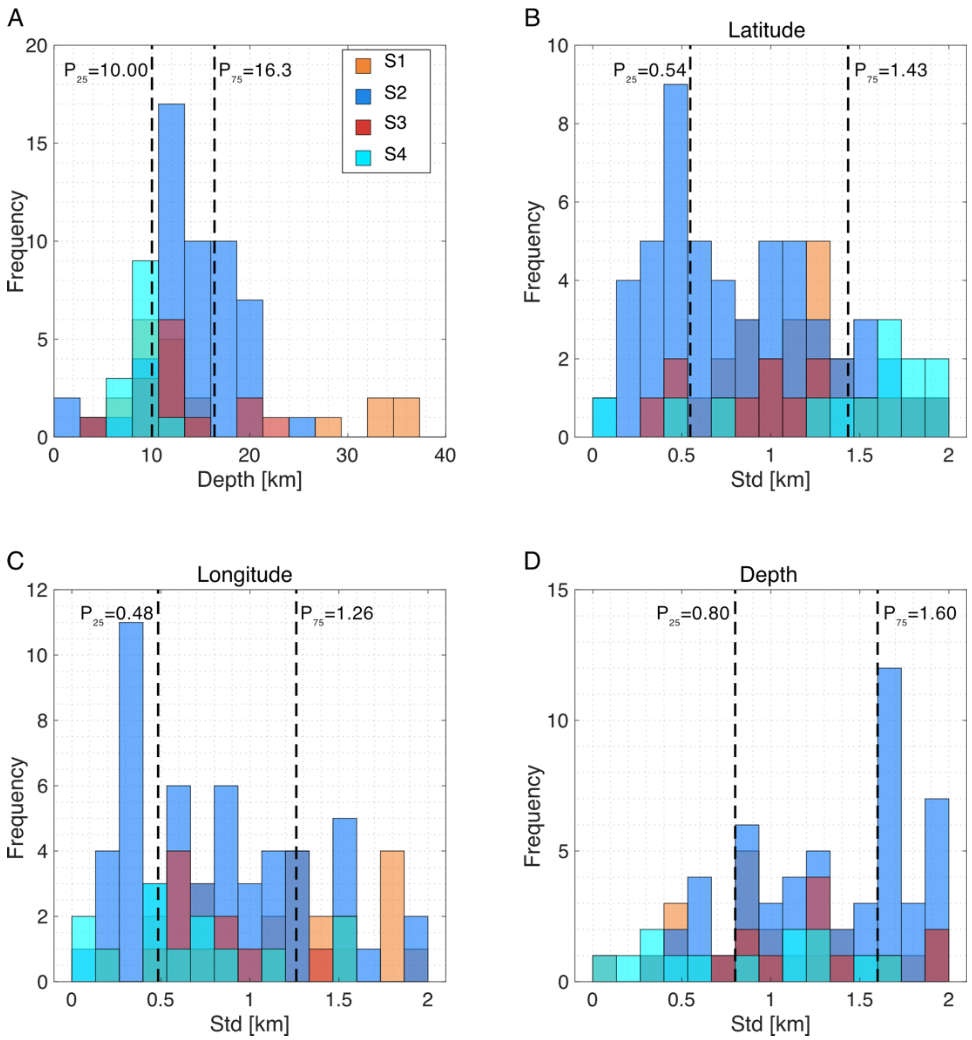

Supplementary Figure 1. Depth and error histograms. (**A**) Histogram of depth of detected tremors for each cluster (S1-S4) described in main text and shown in Fig. 2. Vertical black dashed lines are percentiles 25 and 75 for data of all clusters together. (**B-D**) Histograms of tremor location error for latitude, longitude and depth, respectively. Vertical black dashed lines are percentiles 25 and 75 for data of all clusters in each histogram.

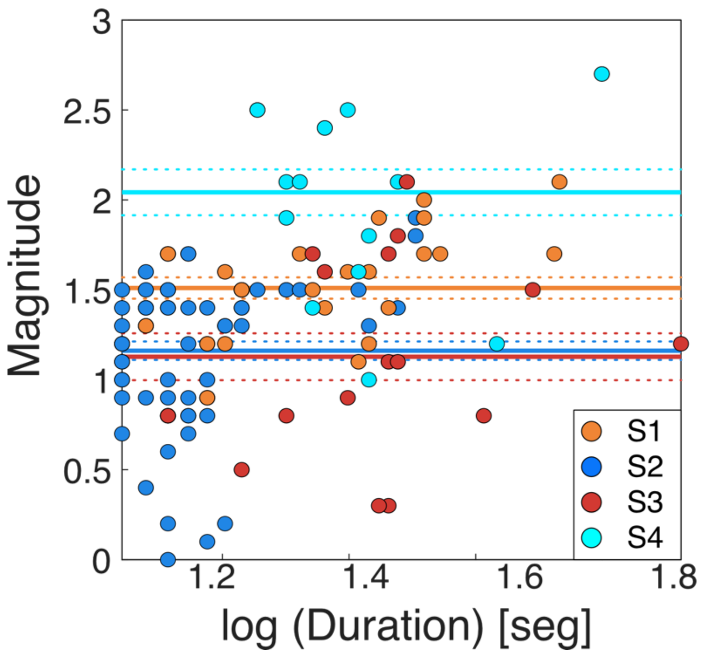

Supplementary Figure 2. Duration vs tremor magnitude. Source parameters for shallow tremors. Solid and dashed lines are mean values of magnitude and uncertainty of the mean, respectively, for each cluster. Colours of symbols are as in Fig. 2B.

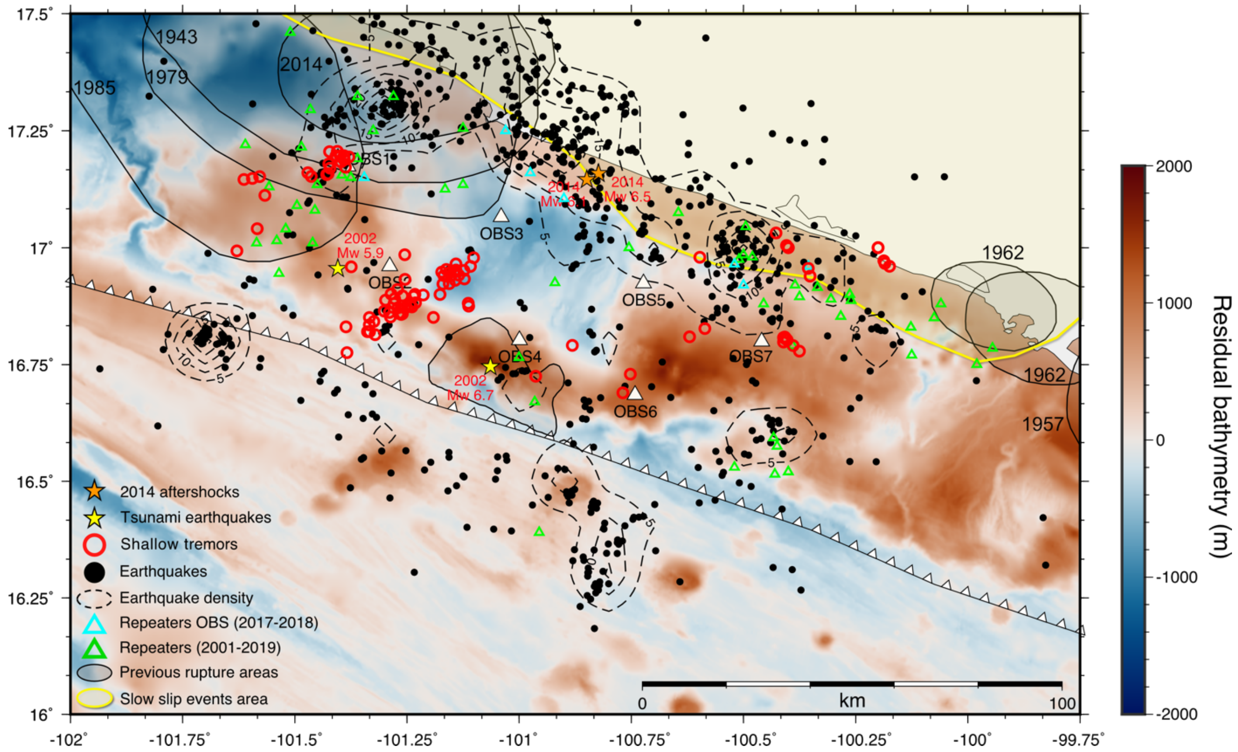

Supplementary Figure 3. Residual bathymetry at the NW-GG. Yellow and orange stars indicate epicentres of the 2002 tsunami earthquakes and aftershocks of the 2014 earthquake, respectively. Red circles, green triangles and black circles are locations of STs, repeaters and earthquakes, respectively. Black dashed lines are contour lines for earthquake density. Grey and yellow areas are past earthquakes ruptures zone[8, 9] and SSEs, respectively.

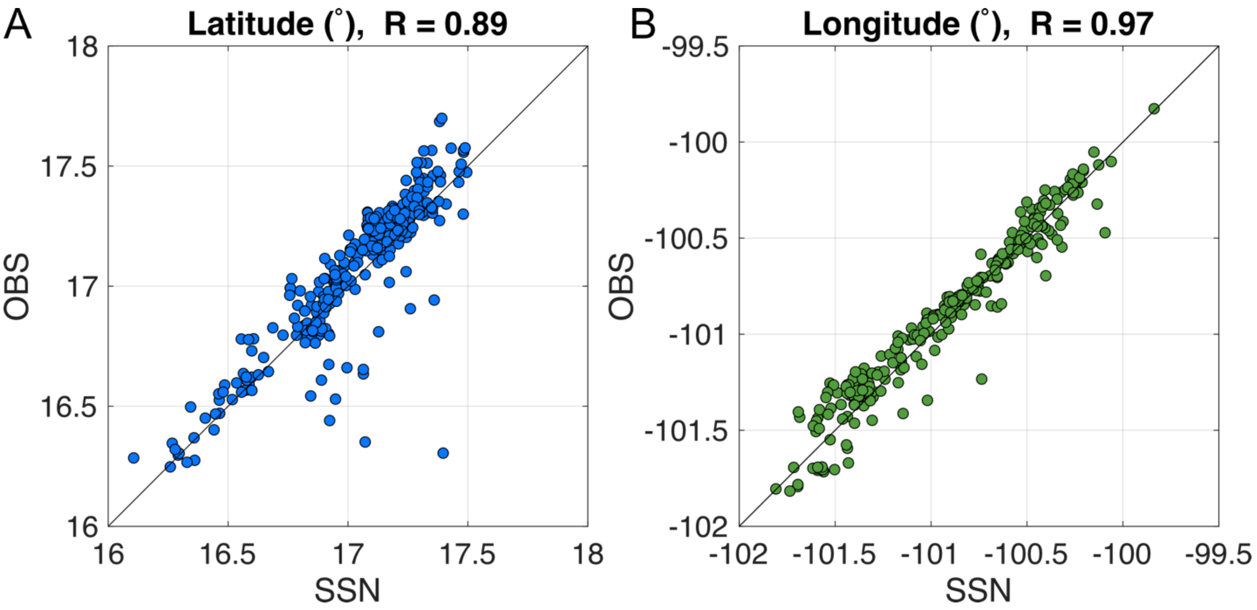

Supplementary Figure 4. Comparison of OBS and SSN earthquake catalogues. OBS catalogue from ocean bottom seismometers and SSN (National Seismological Service) catalogue coming from inland stations in the same time period (2017-2018). (**A**) Latitude for common earthquakes that have been reported in both catalogues. R in the title is the correlation coefficient between the two catalogue observations. Solid black line is a reference for a slope equal to 1. (**B**) same as A for longitude.

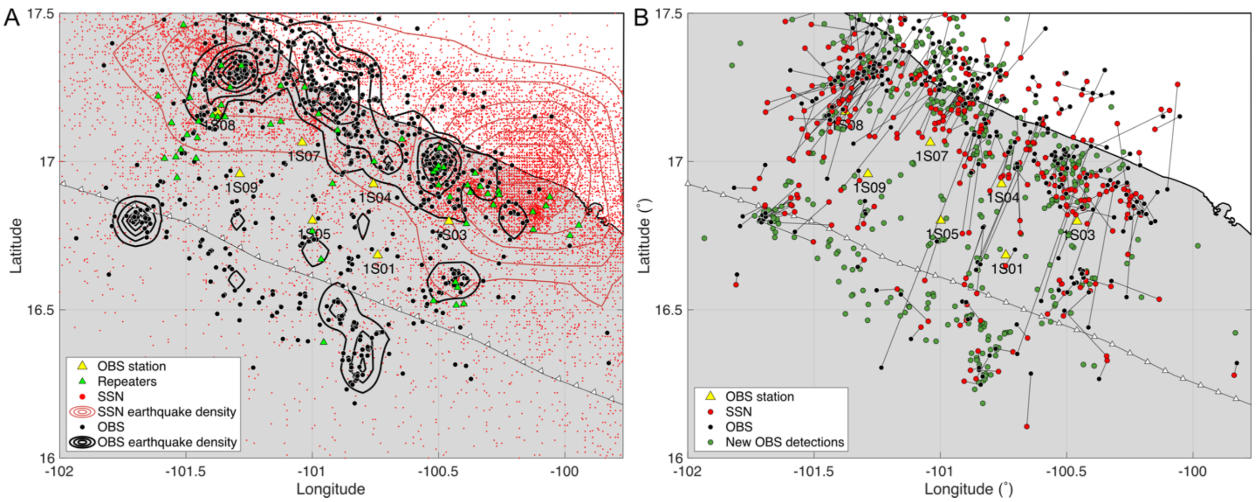

Supplementary Figure 5. Location and density of OBS and SSN earthquake catalogues. (**A**) Black circles and red dots are earthquake locations from the OBS catalogue and from the SSN catalogue, respectively. Black and red contours show the earthquake density for OBS and SSN catalogues, respectively. Green and yellow triangles are repeaters and OBS stations, respectively. (**B**) Location comparison for common earthquakes reported in both catalogues. Earthquake locations from the OBS catalogue shown with black circles are connected with a black line to the corresponding locations from the SSN catalogue shown with red circles. Green circles are earthquakes reported only by the OBS catalogue.

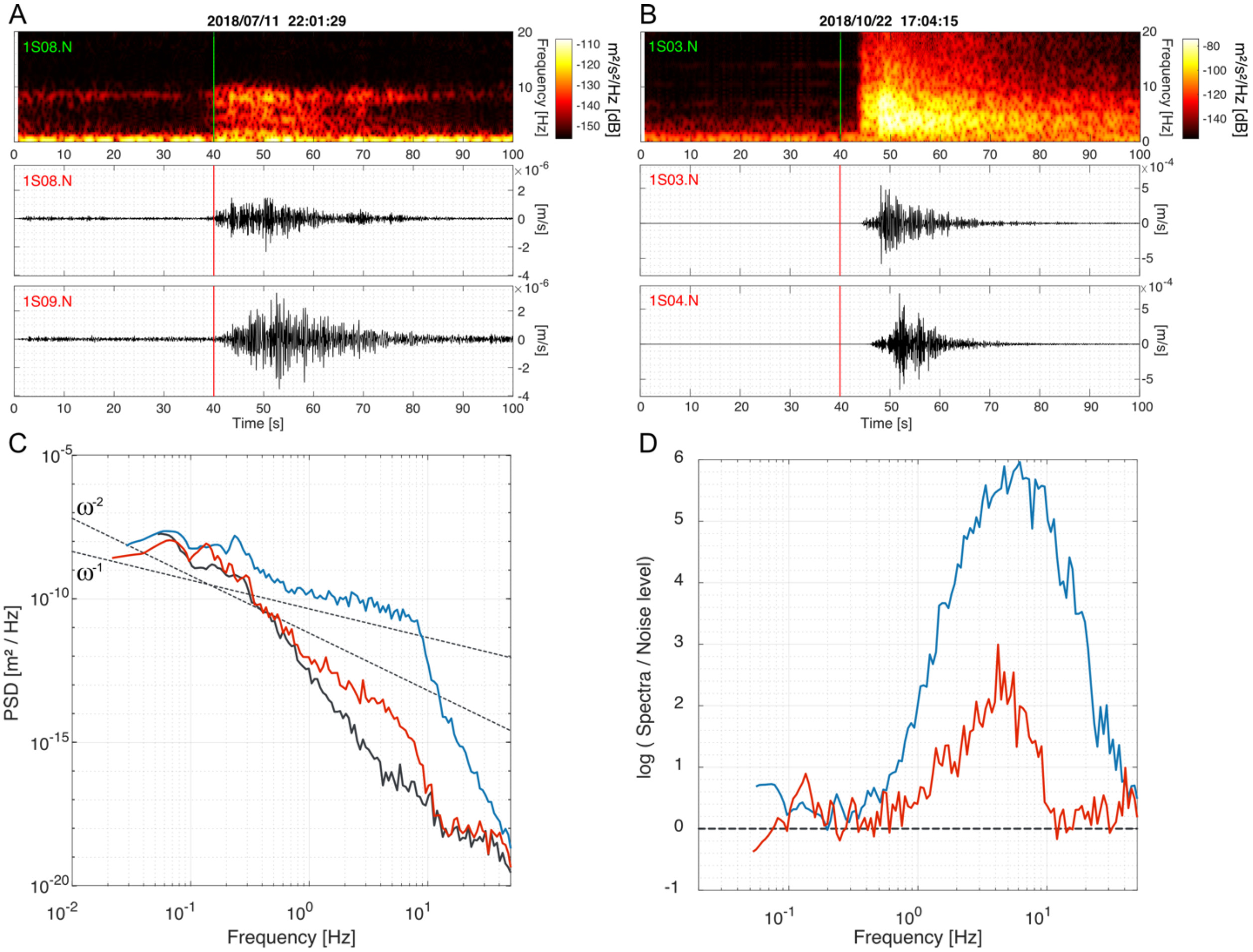

Supplementary Figure 6. Characteristics of tremor and earthquakes. (**A**) Tremor spectrogram and waveform in one horizontal component of two stations. Tremor was detected on 2018/07/11 at 22:01:29. Origin time of tremor is shown with a vertical green line in the spectrogram and red in the seismograms. (**B**) same as A but with an earthquake magnitude 3.2, detected on 2018/10/22 at 14:04:15. Notice that scales in A and B are different. (**C**) Power spectral density (PSD) of displacement records from tremor in A (red) and earthquake in B (blue). Black line is background seismic noise from a time window previous to the occurrence of the tremor. Dashed black lines are references of an ω^{-1} and ω^{-2} slopes. (**D**) Same two spectra from C, normalized with background seismic noise levels from a time window before their occurrence. Dashed black line indicates the level in which the signal to noise ratio is equal to one.

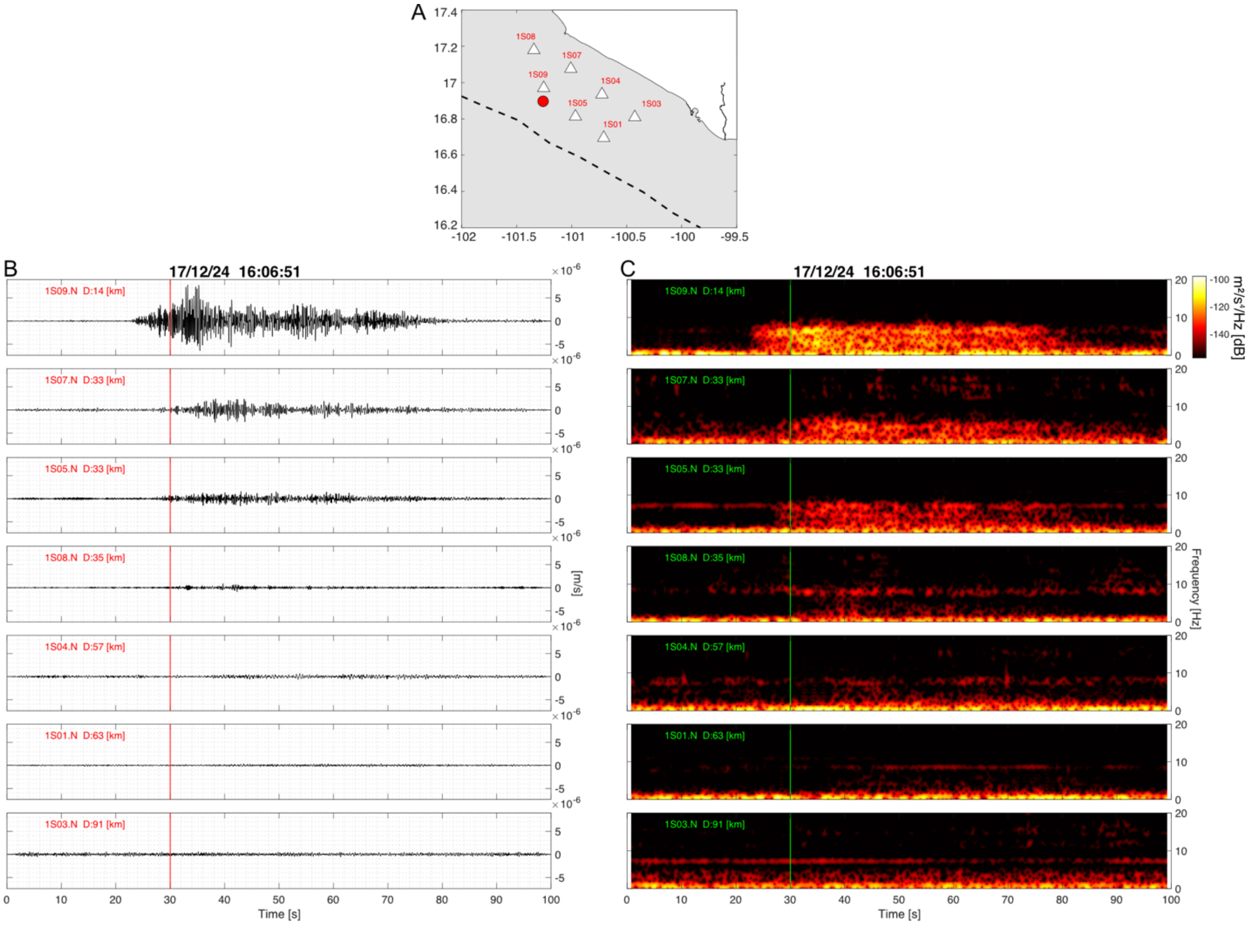

Supplementary Figure 7. Tremor waveform. (**A**) Location of tremor detected on 17/12/24 at 16:06:51 shown with a red circle. White triangles are OBS stations and the dashed black line is the Middle America Trench. (**B**) Waveform of tremor in A, bandpassed filtered (2 - 8 Hz) and seen in one horizontal component of all OBS stations. Vertical red line indicates detection time. Name of stations and their distance to the tremor source are indicated in each waveform. All waveforms have the same vertical scale. As waves travel through the stations array, they attenuate rapidly. (**C**) Spectrogram observed in all the stations, as shown in B. Origin time of tremor is shown with a vertical green line.

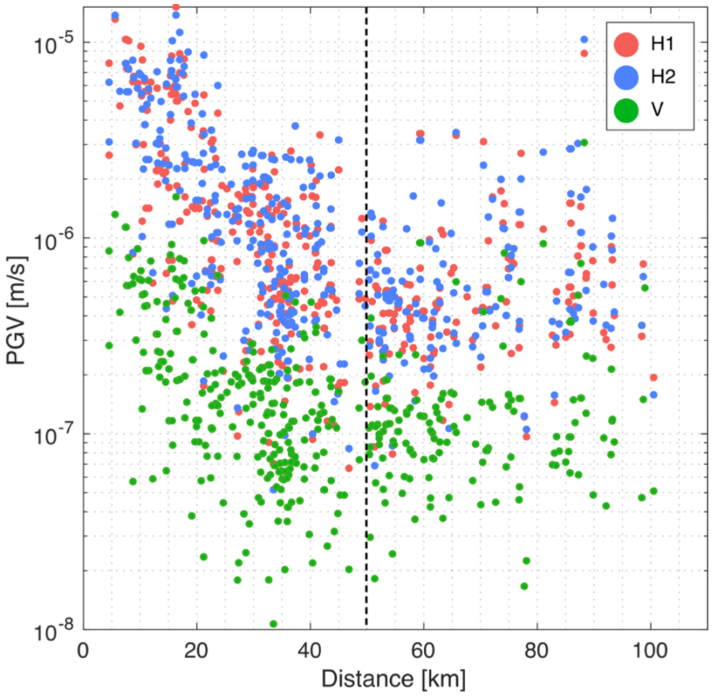

Supplementary Figure 8. Attenuation. Peak ground velocity (PGV) vs distance for tremor signals bandpass filtered between 2-8 Hz. Distance is measure from the epicenter of tremor to each station. Pink and blue circles are PGV values from the two horizontal components and green circles from the vertical component. Due to rapid energy loss, tremor signal is clear when distance between epicenter and station in below 45 km (black dashed line). At greater distances PGV values are mostly from background seismic noise.

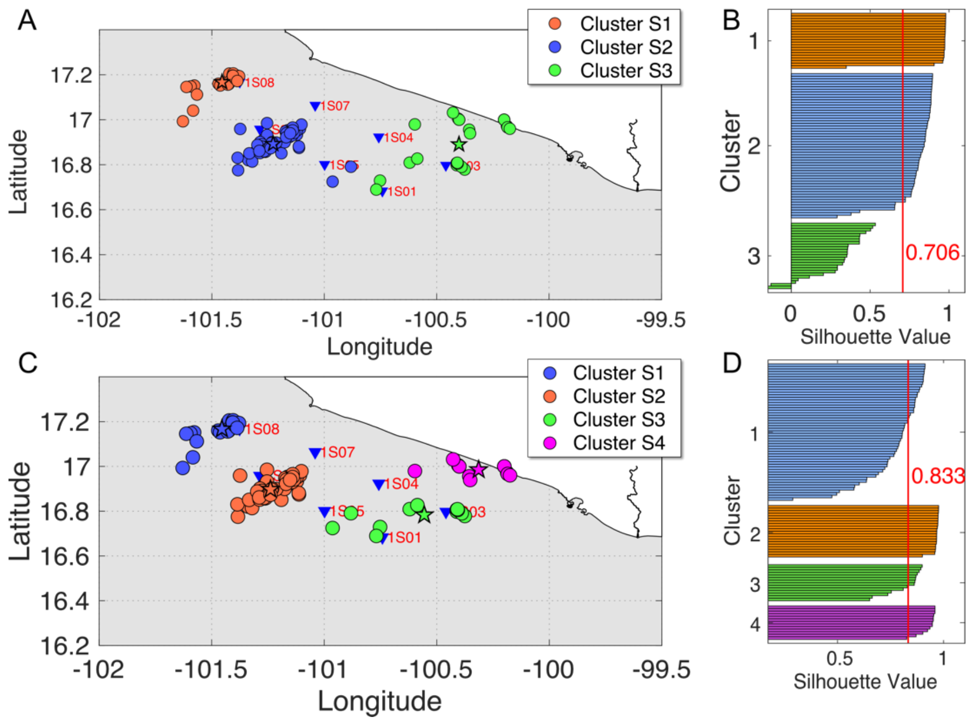

Supplementary Figure 9. K-means clustering for shallow tremors. Two different clustering solutions for shallow tremors, one with 3 clusters (**A**) and the other with 4 clusters (**B**). Clusters members are shown with different colour circles, and inverted blue triangles show OBS locations. (B, C) Silhouette plots for the clustering solutions with 3 clusters and 4 clusters, respectively. Red vertical line shows the mean value of the silhouette values of all shallow tremors.

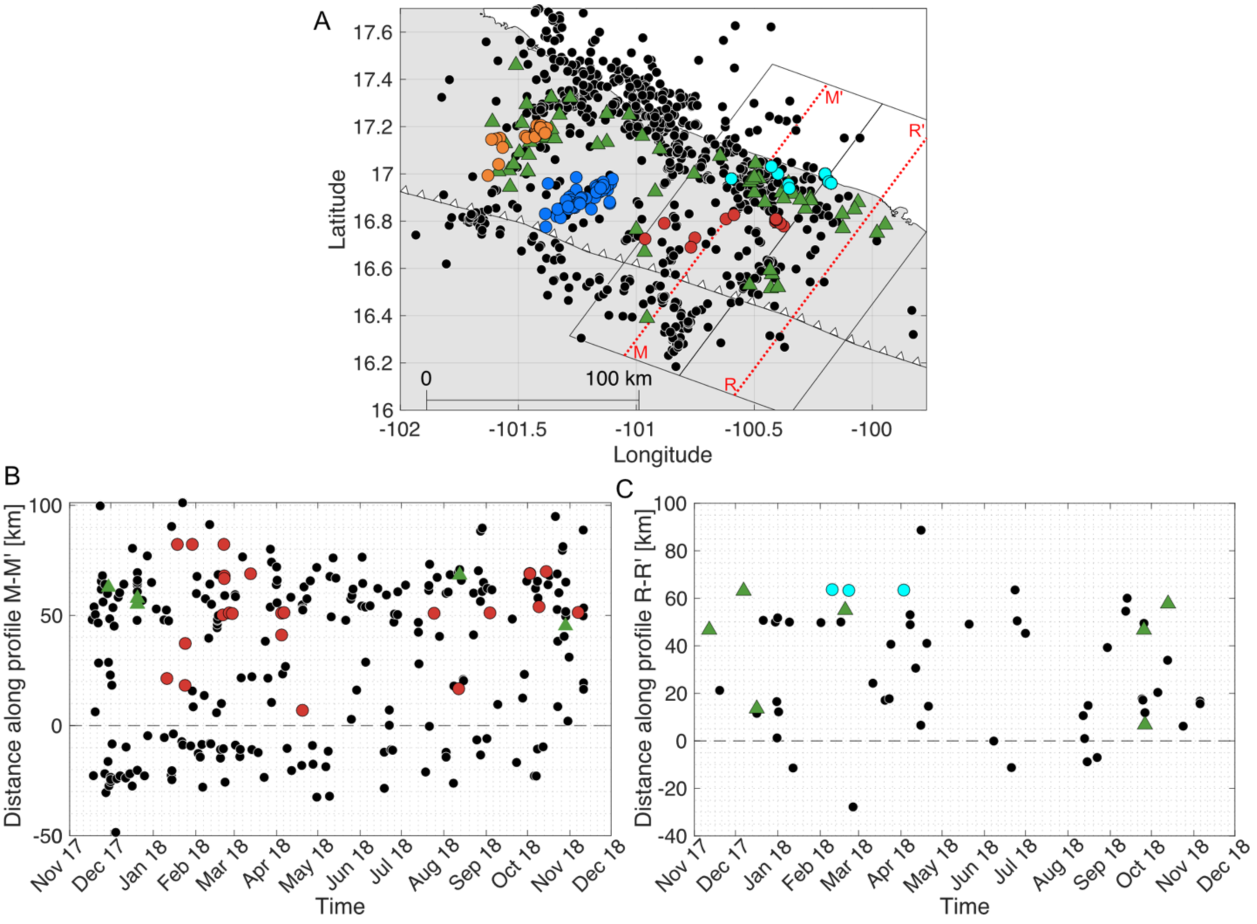

Supplementary Figure 10. Profiles inside the NW-GG. (**A**) Distribution of STs (colour circles) into four main clusters as in Fig. 2A. Green triangles and black circles are repeaters and earthquakes, respectively. Red dotted lines are profiles M-M' and R-R' both inside the NW-GG. Rectangles containing the profiles delimit the seismic events included into B and C. (**B, C**) Time plot vs distance along profiles M-M' and R-R' shown in A. Black dashed line is the location of the trench. Colours and symbols as in A, showing only events occurring during 2017-2018.

Supplementary references:

1. Obana, K. & Kodaira, S. Low-frequency tremors associated with reverse faults in a shallow accretionary prism. *Earth and Planetary Science Letters*, **287**, 169-174 (2009).
2. Sugioka, H. et al. Tsunamigenic potential of the shallow subduction plate boundary inferred from slow seismic slip. *Nature Geoscience*, **5**, 414-418 (2012).
3. Saffer, D. & Wallace, L. The frictional, hydrologic, metamorphic and thermal habitat of shallow slow earthquakes. *Nature Geoscience*, **8**, 594-600 (2015).
4. Brune, J. N. Tectonic stress and the spectra of seismic shear waves from earthquakes. *Journal of Geophysical Research, 75*(26), 4997-5009 (1970).
5. Okal, E. A. The generation of T waves by earthquakes. *Advances in Geophysics*, **49**, 1-65 (2008)
6. David, A. & Sergi, V. K-means++: The advantages of careful seeding. *SODA '07: Proceedings of the Eighteenth Annual ACM-SIAM Symposium on Discrete Algorithms*, New Orleans, Lousiana, 7-9 January 2007, 1027-1035 (2007).
7. Kaufman L. & Rousseeuw P. J. *Finding Groups in Data: An Introduction to Cluster Analysis*. Hoboken NJ: John Wiley & Sons Inc (1990).
8. Flores-Ibarra, K. "Estudio de la fuente compleja del sismo 18 de abril del 2002 (Mw 6.7), fuera de la costa de Guerrero", thesis, Universidad Nacional Autónoma de México, Mexico City (2018).
9. Kostoglodov, V. & Pacheco, J. *Cien Años de Sismicidad en México,* retrieved from http://usuarios.geofisica.unam.mx/vladimir/sismos/100a%F1os.html (last accessed March 2020) (1999)
