# Supplementary material for: Shallow slow earthquakes to decipher future catastrophic earthquakes in the Guerrero seismic gap
Source: Nat Commun. 2021 Jun 28;12:3976. doi: 10.1038/s41467-021-24210-9 (PMC8239025; doi:10.1038/s41467-021-24210-9)
Supplement: Supplementary file 2 — Description of Additional Supplementary Files [file 41467_2021_24210_MOESM2_ESM.pdf]

Description of Additional Supplementary Files

File name: Supplementary Data 1

Description: Shallow tremor catalogue detected by OBS data. Origin time is indicated in UTC time.

File name: Supplementary Data 2

Description: Earthquake catalogue detected by OBS data. Origin time is indicated in UTC time.

File name: Supplementary Data 3

Description: Repeaters catalogue detected in Guerrero. Origin time is indicated in UTC time.
